# The efficacy of vacuum-ultraviolet light disinfection of some common environmental pathogens

**DOI:** 10.1186/s12879-020-4847-9

**Published:** 2020-02-11

**Authors:** Wai Szeto, W. C. Yam, Haibao Huang, Dennis Y. C. Leung

**Affiliations:** 1 Department of Mechanical Engineering, The University of Hong Kong, Hong Kong, China; 2 Department of Microbiology, The University of Hong Kong, Hong Kong, China; 3 School of Environmental Science and Engineering, Sun Yat-sen University, Guangzhou, China

**Keywords:** Disinfection, Microorganism, Ozone, VUV, IAQ, Influenza, Tuberculosis, ESBL, MRSA

## Abstract

**Background:**

This study is to elucidate the disinfection effect of ozone producing low-pressure Hg vapor lamps against human pathogens. Ozone producing low-pressure Hg vapor lamps emit mainly 254 nm ultraviolet light C (UVC) with about 10% power of Vacuum-ultraviolet (VUV) light at 185 nm. The combination of UVC and VUV can inactivate airborne pathogens by disrupting the genetic materials or generation of reactive oxygen species, respectively. In this study, inactivation of common bacteria including *Escherichia coli* ATCC25922 (*E. coli*), Extended Spectrum Beta-Lactamase-producing *E. coli* (ESBL), Methicillin-resistant *Staphylococcus aureus* (MRSA) and *Mycobacterium tuberculosis* (MTB), and that of influenza A viruses H1N1 and H3N2 under the radiation from ozone producing low-pressure Hg vapor lamps was examined. Log reduction values at different treatment durations were determined.

**Methods:**

In vitro tests were carried out. Various bacterium and virus suspensions were added onto nitrocellulose filter papers and subjected to the illumination from ozone producing low-pressure Hg vapor lamps. The extents of pathogen inactivation at different illumination times were investigated by conducting a series of experiments with increasing duration of illumination. log10 reduction in CFU/ml and reduction at log10(TCID_50_) were respectively measured for bacteria and viruses. The disinfection effectiveness of this type of lamps against the pathogens under the environment with a moderate barrier to light was therefore evaluated.

**Results:**

Ozone producing low-pressure Hg vapor lamp successfully inactivated these human pathogens. Nevertheless, among these pathogens, disinfection of MTB required more intense treatment. In the best tested situation, 3-log10 inactivation of pathogens can be achieved with ≤10 min of VUV treatment except MTB which needed about 20 min. This demonstrated the high resistance against UV disinfection of MTB.

**Conclusions:**

Following the criteria that valid germicidal results can be reflected with 3-log10 inactivation for bacteria, 4-log10 inactivation for viruses and 5-log10 inactivation for MTB, most of the bacteria required ≤10 min of VUV treatment, 20 min for the influenza viruses while MTB needed about 30 min VUV treatment. This indicated that VUV light is an effective approach against different environmental microorganisms.

## Background

Indoor air quality (IAQ) has a significant influence on health, comfort and well-being of building occupants. It has been demonstrated that poor IAQ could jeopardize health and well-being, which in turn will affect the quality of work and ultimately lower the productivity of workers [1].

One major source of indoor air pollution is the presence of micro-organisms, which could cause even more serious problems than some organic and inorganic air contaminants. This is particularly more phenomenal in cases of inadequate ventilation, as the condensation in ventilation system can act as a breeding ground for harmful bacteria which are dispensed through the ventilation ducts. Environmental airborne bacteria such as *Pseudomonas aeruginosa*, *Streptomyces albus*, *Bacillus subtilis* and complex populations of micro-organisms within normal flora were all etiological agents to hypersensitivity pulmonary diseases. Several additional infectious agents such as *Legionella pneumophila* and *Mycobacterium tuberculosis* (MTB) pose even more grave concerns to the IAQ, as these airborne pathogenic bacteria are known to cause severe illness in humans. Meanwhile, viruses such as influenza virus were originally thought to be only transmitted from person to person via aerosols of body fluids. However, in a recent study conducted by Weistein et al. [2], the production of infectious droplet nuclei of diameter < 5 μm could remain suspended and disseminated by air current to infect a susceptible host. A good and reliable disinfection system, therefore, is required to disinfect the airborne microorganisms in order to maintain good IAQ.

Adopting vacuum-UV (VUV) lamps, for instance, the ozone producing low-pressure Hg vapor lamps, can be an effective mean of disinfecting the airborne microorganisms. Many existing infection control products use low pressure mercury vapor lamps as light source. This is a source of high energy photons with low cost. Recently, pulsed xenon light source technology emitting a broad spectrum (200-300 nm) of UV light is an emerging alternative to low pressure mercury vapor lamps that allows much faster surface disinfection because of the high peak power [3]. Nevertheless, the pulsed nature of this technology would limit its use in continuous air disinfection system. Electrical discharge of low pressure mercury vapor mainly emits 254 nm ultraviolet light C (UVC) and 185 nm VUV light. However, existing products mainly use the lamps with doped quartz envelope that absorbs 185 nm photons to prevent the formation of potentially dangerous ozone. Nevertheless, ozone is also a powerful disinfectant and the valuable disinfection opportunity of the 185 nm VUV light becomes waste heat.

Ozone is an issue that bothers on safety if it remains in the output of an air treatment system. However, ozone can be easily destroyed before leaving the air treatment system if proper catalyst is adopted [4, 5]. Also, some photocatalysts can utilize and destroy ozone in addition to its photocatalytic activity [6].

The 254 nm UVC light adopted in conventional infection control products can disinfect the illuminated objects since the 254 nm radiation can disrupt the genetic materials of airborne pathogens and render them inviable [7].VUV has an even stronger ionizing power than UVC light and can generate high concentration reactive species such as ozone and OH radicals [7]. In other words, apart from direct illumination, VUV can inactivate bacterial growth by the radicals generated during VUV irradiation. Therefore, adopting VUV lamps can enhance the air disinfection capability of air cleaning systems. A previous study [4] conducted by Huang et al. demonstrated that 64% toluene removal with VUV irradiation alone and the use of photocatalyst enhanced the toluene removal from 64 to 82%. The experiment adopting UVC lamps and the use of photocatalyst removed only 14% of toluene. The result demonstrated that VUV light could be an effective measure for chemical degradation in ventilation systems. When it comes to disinfection, extensive research has been carried out on UVC light and effective destruction of both airborne [8–20] and other human pathogens [21–29] has been shown. Nevertheless, disinfection using VUV light has attracted very little attention. This would be caused by the relative low prevalence of VUV light sources. Kim et al. [30] found that the disinfection time required to attain the same extent of inactivation of aerosolized MS2 bacteriophage, using low pressure mercury vapor lamps with both 254 nm UVC and 185 nm VUV output was much shorter than the lamps with 254 nm UVC only. The disinfection time of ozone only (without UV) process at ozone concentrations equivalent to the ozone level generated by the mercury vapor lamps was also significantly faster than using lamps with 254 nm emission only. Besides, Huang et al. [4] reported the inactivation of *E coli* by low pressure mercury vapor lamps. Additionally, some researchers tested the disinfection of water with VUV light and it was reported that the efficiency was quite low compared to disinfection with UVC light [31, 32]. The reason is due to the low penetration power of VUV light in water [33]. Moreover, the disinfection of human pathogens by VUV light was rarely reported. In our opinion, only Christofi et al. [34] reported the disinfection of the microbial films of 3 types of pathogenic bacteria using ozone producing low-pressure Hg vapor lamps. Therefore, the effect of VUV light against human pathogens is yet to be elucidated. In this study, we evaluated the germicidal effect of VUV light on common bacteria including *Escherichia coli* ATCC25922 (*E. coli*), Extended Spectrum Beta-Lactamase-producing *E. coli* (ESBL), Methicillin-resistant *Staphylococcus aureus* (MRSA) and *Mycobacterium tuberculosis* (MTB), and that on influenza viruses H1N1 and H3N2. Influenza viruses and MTB are inherent airborne pathogens while *E. coli* ATCC25922 is always the first indicator organism to monitor disinfection efficacy. The more drug resistant ESBL and MRSA were chosen as examples to monitor disinfection efficacy on human pathogens. Some suspensions of these bacteria and viruses were absorbed into nitrocellulose filter papers during the experiments and the disinfection under the environment with a moderate barrier to light was evaluated.

## Methods

### UV irradiation

**Fig. 1:**
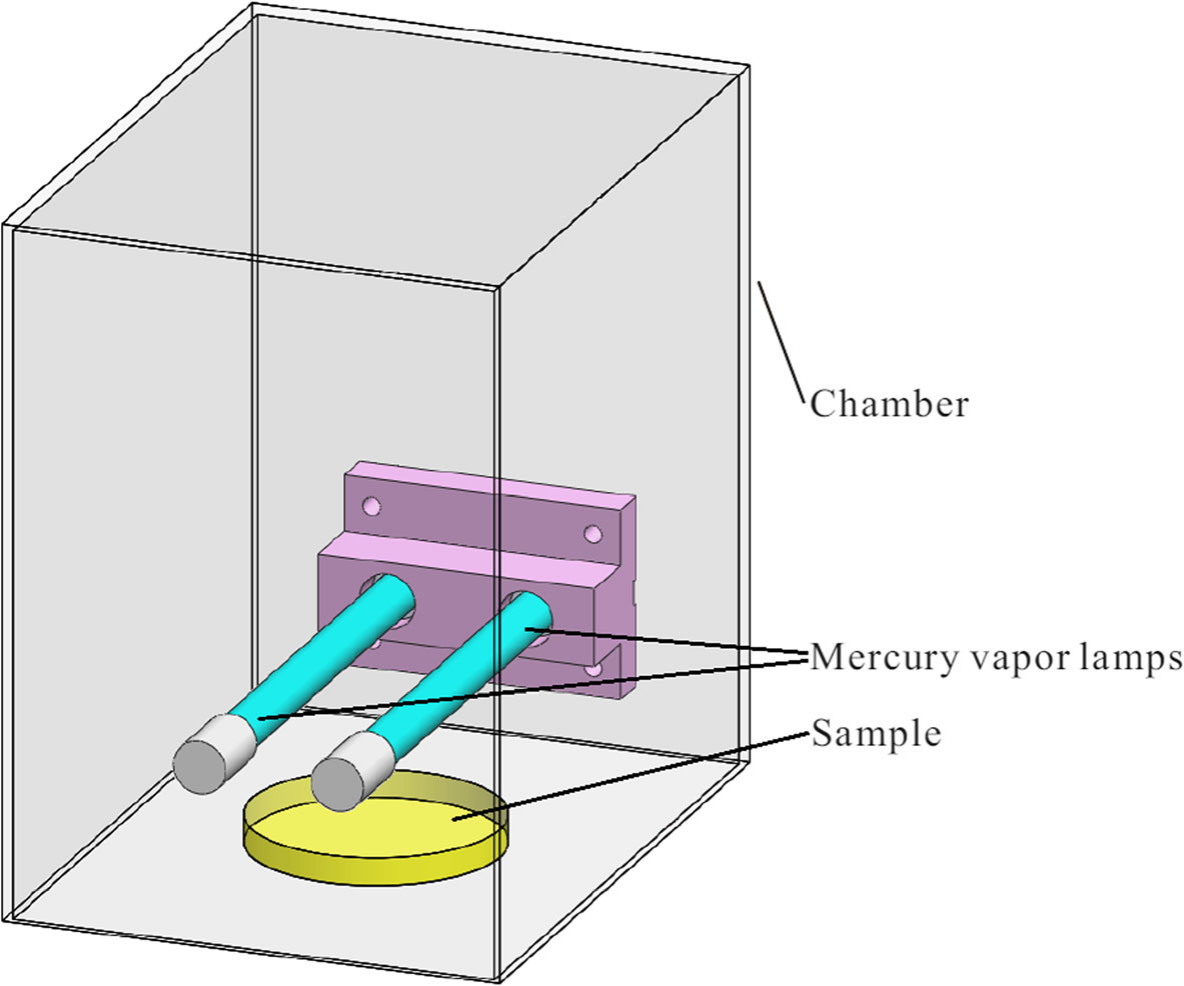
The VUV illumination experiment

To evaluate the biocidal effect of VUV light, bacteria and viruses were irradiated with a pair of hot cathode low pressure mercury vapor lamps. The lamps were 10 W, U-VIX brand, ZW10D15Y, ozone generating. The distance between the light source and the microorganisms was approximately 5 cm and the UV intensities at 254 nm and 185 nm, respectively measured by a ZDZ-1 UV-C meter and an ILT1400 radiometer were 21 and 2.3 mW/cm^2^, respectively. To reduce the leakage of UV light and lamp-generated ozone to the surrounding, the lamps and the microorganisms under test were contained in a metal chamber during the experiments as shown in Fig. 1.

### Bacterial strains and inoculum preparation

Following procedures were used to prepare bacterial samples for UV irradiation experiments.

#### *Escherichia coli* ATCC25922 (*E. coli*), extended Spectrum Beta-Lactamase-producing *E. coli* (ESBL) and methicillin-resistant *Staphylococcus aureus* (MRSA)

*Escherichia coli* strain ATCC25922 (*E. coli*), fully susceptible to most antibiotics, was purchased from American Type Culture Collection (ATCC). Methicillin-resistant *Staphylococcus aureus* strain QC 5618 (MRSA) was provided as a Proficiency Program of Central Public Health Laboratory, Colindale, UK. Extended Spectrum Beta-Lactamase-producing *E. coli* strain MM1604 (ESBL) was provided as a Proficiency Program of Central Public Health Laboratory Service, Department of Health, Hong Kong.

*E. coli* and MRSA were inoculated onto Mueller-Hinton agar (BD Bioscience, CA, USA) plates and incubated overnight at 37 °C to yield single colonies. Overnight cultures were prepared by inoculating single colonies of each bacterial strain into Brain Heart Infusion (BHI) broth (BD Bioscience, CA, USA). Bacterial suspension at early exponential phase was inoculated into BHI broth at 37 °C for 2 h. The concentration of the bacterial suspension was then visually adjusted to McFarland standard 0.5. Test suspension was prepared by diluting the 0.5 McFarland standard inoculum by 10-fold and 100-fold. Actual bacterial count was calculated by back titration of the inoculum suspension. Purity of MRSA was checked by ChromID® MRSA agar plate (BioMérieux SA, France) and the purities of *E.coli* and ESBL-producing *E. coli* were confirmed by MacConkey agar plate (Oxoid™, Thermo Scientific, Massachusetts, United States).

#### *Mycobacterium tuberculosis* (MTB)

MTB H37Rv (ATCC27294) was selected as the model organism. Due to the infectivity and the risk of handling MTB, the experiments were conducted in the Biosafety Level-3 Laboratory of The University of Hong Kong.

MTB was first inoculated onto non-selective Middlebrook 7H11 agar (BD Bioscience, CA, USA) supplemented with 10% Oleic acid-Albumin-Dextrose-Catalase (OADC) and incubated at 37 °C with 5% CO_2_ until single colonies were obtained. Mycobacterial colonies were resuspended into glass-bead Phospate-Buffered Saline with 0.1% Tween 80. Inoculum was vortexed for 30 s to homogenize the bacterial suspension. Bacterial concentration was then adjusted to optical density at 600 nm = 0.15–0.17, which is equivalent to 0.5 McFarland standard. Two test suspensions were prepared, which were 0.5 McFarland standard inoculum and 10-fold diluted 0.5 McFarland suspensions. Actual MTB count was calculated by back titration of the inoculum suspension on Middlebrook 7H11 agar. Purity of MTB was checked by culturing the inoculums on blood agar to ensure no fungal and bacterial contamination, and on non-selective Middlebrook 7H11 agar to ensure there was no contamination by nontuberculous mycobacteria.

### Virus strains and cell lines

#### H1N1 and H3N2

Following procedures were used to prepare viral samples for UV irradiation experiments.

H1N1 was isolated from the first swine flu patient in Hong Kong in 2009 by the Department of Microbiology, The University of Hong Kong. H3N2, a seasonal flu in Hong Kong, was generously provided by Prof. H.L. Chen, Department of Microbiology, The University of Hong Kong. MDCK (Madin-Darby canine kidney) cell line provided by CDC, USA, was used to cultivate H1N1 and H3N2 viruses.

Both seasonal influenza A viruses were cultured in MDCK cells in MEM (GiBCO) supplemented with TPCK-trypsin (Sigma-Aldrich, MO, USA). Virus-infected cells were harvested when almost all MDCK cells exhibited cytopathic effects. Infected cells and the conditioned media underwent one freeze-thaw cycle to release viral particles. The suspension was then centrifuged at 3000 rpm for 5 min, and supernatant containing viral particles was collected. Tissue culture infective dose 50 (TCID_50_) was determined in a 96-well tissue culture plate using Reed Muench method. Virus stock was stored at − 80 °C prior usage.

### UV disinfection experiments

#### VUV disinfection experiments of *E. coli*, ESBL and MRSA

To analyze the bactericidal effect of VUV light, 2 mL of bacterial suspension was added onto the nitrocellulose filter and irradiated by VUV for 2, 5, 10 and 15 min at a distance of 5 cm at 25 °C. This distance was selected based on the consideration of the time of disinfection and temperature rise of the agar during the course of experiments. As each experiment was carried out inside a Level-2 Biosafety Cabinet, the 2 mL added suspension was carefully adjusted so that the filter remained moisted at the end of irradiation as dryness will reduce the viable count recovered from the filter.

The illuminated bacterial suspension and the nitrocellulose filter were vigorously washed by 10 mL Phosphate-buffered saline (PBS). The suspension was then serially diluted with PBS from 10^0^ to 10^− 4^, and 100 μL of each of the serially diluted bacterial suspensions was spread onto a Mueller-Hinton agar plate. Meanwhile, bacterial test suspensions without VUV illumination were spread onto Mueller Hinton agar to obtain the initial colony-forming units (CFU) before the use of VUV light disinfection as control.

All Mueller-Hinton agar plates were incubated overnight at 37 °C. The resultant CFU in each test suspension reflected the viable bacterial count after different disinfection durations. The disinfection assay was carried out in triplicate for each bacterial strain.

#### VUV disinfection experiments of *Mycobacterium tuberculosis*

To investigate the minimum time required by VUV light for optimal MTB disinfection, test sets were used in which 2 mL concentration-adjusted MTB inoculums, added onto nitrocellulose filter papers, were illuminated by VUV for 10, 20, 30 and 45 min.

The illuminated bacterial suspension and nitrocellulose filter were vigorously washed by 10 mL PBS, and the suspension was serially diluted (10^0^–10^− 4^). A total of 100 μL of each diluted bacterial suspension was spread onto selective Middlebrook 7H11 agar supplemented with 10% oleic albumin dextrose (OADC), 200,000 unit/L Polymyxin B, 50 mg/L Carbenicillin, 10 mg/L Amphotericin B and 20 mg/L Trimethoprim Lactate. Bacterial inoculum without VUV illumination was used as MTB growth control and to determine the original viable bacterial count. Each test set was conducted in triplicate.

#### VUV disinfection experiments of influenza viruses H1N1 and H3N2

To analyze the virucidal effect of VUV light, 2 mL virus samples at ~ 1 × 10^6^ TCID_50_/mL were added onto nitrocellulose filter papers and irradiated by vacuum ultraviolet light (VUV) for 5, 10, 15 and 20 min at an illumination distance of 5 cm at 25 °C. The illuminated viral suspension and nitrocellulose filter were vigorously washed, and the suspension was then serially diluted (10^0^–10^− 8^) by Minimum Essential Medium (MEM) supplement with TPCK-trypsin. Each diluted sample was used to infect Madin-Darby Canine Kidney (MDCK) cells in the presence of TPCK-trypsin at 37 °C for 3 days. The end point of cytopathic effects (CPE) as small, round and degeneration was recorded. Virus sample without VUV illumination was used to infect MDCK as positive control and to determine the original viral load. Each test was conducted in triplicate.

### Data analysis

For bacteria, log10 reduction of viable bacterial count in CFU/mL was calculated by comparing control and post irradiation filters.

For influenza viruses, reductions at log10 (TCID_50_) was calculated similarly.

For each test, outliers were removed by Dixon’s Q test at 95% significance level. The resultant log10 reduction in CFU/ml of each bacterial strain and the resultant log10 reduction in TCID_50_ for each viral strain were plotted against disinfection durations, and error bars showing the data of the experiments that deviate from the corresponding mean value were also provided. MS Excel was used in all calculations and graph generation. A spreadsheet file containing raw data and intermediate calculations is provided in as a supplementary information file.

## Results

### *Escherichia coli* ATCC25922 (*E. coli*)

**Fig. 2:**
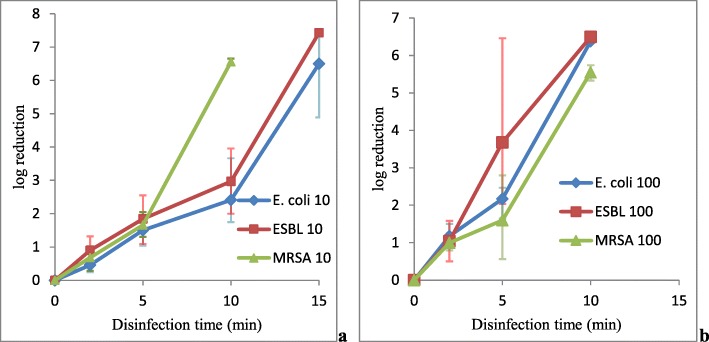
VUV light disinfection against *E. coli*, ESBL and MRSA*.* Both 10-fold (**a**) and 100-fold (**b**) diluted 0.5 McFarland standard inoculums of *E. coli* (denoted by *E. coli* with the dilution ratio behind), ESBL (denoted by ESBL with the dilution ratio behind) and MRSA (denoted by MRSA with the dilution ratio behind) were subjected to VUV light disinfection. The log10 (CFU/mL reduction) were plotted against the time of disinfection. Data were plotted as the means of triplicate biological replicates ±error

Initial inoculum sizes for *E. coli* in 10-fold diluted and 100-fold diluted 0.5 McFarland standard inoculums across triplicate experiment sets, presented in the Additional file 1 as Expt. 1 and Expt. 2, were (1.9 ± 0.6) × 10^7^ CFU/mL and (2.4 ± 0.2) × 10^6^ CFU/mL, respectively. At 10 min VUV light disinfection, the device was able to produce at least 6-log10 reduction in viable bacterial count for 100-fold diluted 0.5 McFarland standard inoculum. However, 10 min VUV light disinfection for 10-fold diluted 0.5 McFarland standard inoculum can only achieve a borderline to insufficient bactericidal activity with an average 2.4-log10 growth reduction and 99.57% inhibition of bacterial growth (Fig. 2a and b). The results suggested that VUV light disinfection is much more effective against lower *E. coli* bacterial concentration. At 15 min disinfection, complete inhibition of bacterial growth was also observed in 10-fold diluted 0.5 McFarland standard inoculum, resulting in at least 6-log10 growth reduction (Fig. 2a and b).

### Extended Spectrum Beta-Lactamase-producing *E. coli* (ESBL)

Initial bacterial counts of ESBL for 10-fold diluted and 100-fold diluted 0.5 McFarland standard inoculums across triple experimental sets, presented in the Additional file 1 as Expt. 3 and Expt. 4, were (2.7 ± 0.3) × 10^7^ CFU/mL and (3.2 ± 0.7) × 10^6^ CFU/mL, respectively. It was observed that after 15-min disinfection, both 10-fold diluted and 100-fold diluted 0.5 McFarland standard inoculums were able to achieve complete inhibition of bacterial growth, resulting in at least 6-log10 growth reduction (Fig. 2a and b). However, at 10-min of disinfection time, although, the device was able to produce at least 6-log10 reduction of bacterial growth for the 100-fold diluted inoculum, VUV light was only able to produce a borderline to insufficient bactericidal effect for the 10-fold diluted 0.5 McFarland standard inoculum. The test only demonstrated an average of 2.96-log10 reduction with 99.63% growth inhibition. The results have demonstrated that VUV light is more effective against a lower concentration of ESBL*.*

### Methicillin-resistant *Staphylococcus aureus* (MRSA)

Initial bacterial counts of MRSA for 10-fold diluted and 100-fold diluted 0.5 McFarland standard inoculums across triple experiment sets, presented in the Additional file 1 as Expt. 5 and Expt. 6, were (3.7 ± 0.9) × 10^6^ CFU/mL and (3.8 ± 1.7) × 10^5^ CFU/mL, respectively. At 10 min of VUV light disinfection, the bacteria of the 10-fold diluted and the 100-fold diluted 0.5 McFarland standard inoculums were completely inhibited, resulting in at least 5-log10 growth reduction (Fig. 2a and b).

### *Mycobacterium tuberculosis* (MTB)

As defined in previous sections, disinfection time against bacteria was considered sufficient when a minimum 3-log10 reduction of viable bacterial count was observed. For mycobactericidal activity, a 5-log10 reduction in viable bacterial load is required due to the highly infectious nature of MTB. In other words, a minimum of 5-log10 viable bacterial load would be required for a valid experimental set. The average bacterial concentration for McFarland standard 0.5 MTB inoculum was only (3–5) × 10^6^ CFU/mL according to our previous experiments (data not shown). When the bacterial inoculum was diluted by 100-fold, the bacterial concentration would only be around 10^4^ CFU/mL. The bacterial load could be too low and it was incapable of illustrating 5-log10 growth reduction. The experiment was therefore conducted with a higher bacterial concentration and more detailed disinfection time as compared to the tests of other bacteria. 0.5 McFarland standard and 10-fold diluted 0.5 McFarland standard inoculums were used and irradiated by VUV for 10, 20, 30 and 45 min. Initial bacterial counts for 0.5 McFarland standard and the 10-fold diluted 0.5 McFarland standard MTB inoculums were (4.4 ± 1.7) × 10^6^ CFU/mL and (1.2 ± 0.2) × 10^5^ CFU/mL, respectively, presented in the Additional file 1 as Expt. 7 and Expt. 8.

**Fig. 3:**
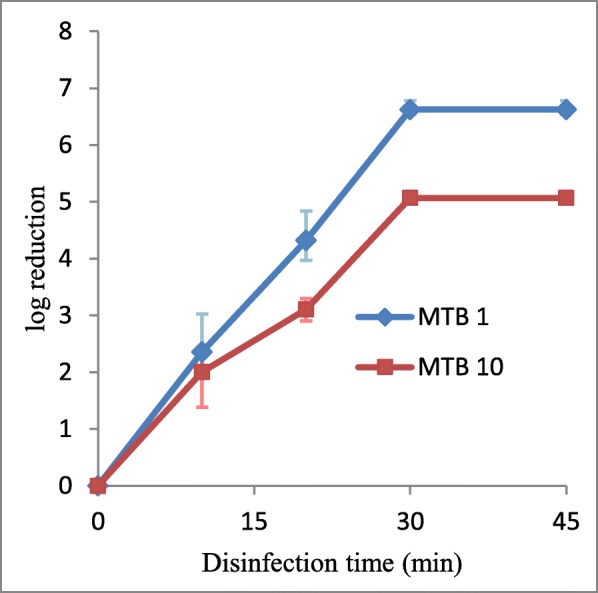
VUV light disinfection against MTB. The experimental sets were conducted on 0.5 McFarland standard inoculum (denoted by MTB 1) and 10-fold diluted 0.5 McFarland standard inoculum (denoted by MTB 10). The log10 (CFU/mL reduction) were plotted against the time of disinfection. Data were plotted as the means of triplicate biological replicates ±error

Gradual reduction in bacterial count was observed with prolonged VUV disinfection time. Complete inhibition of bacterial growth was observed after 30 min VUV light disinfection. At 20 min VUV illumination, VUV light was able to produce an average of 4-log10 and 3.6-log10 reduction in 0.5 McFarland standard and the 10-fold diluted 0.5 McFarland standard inoculums, respectively (Fig. 3).

In the present study, we have demonstrated that VUV light disinfection can achieve complete inactivation of MTB growth after 30 min disinfection regardless of the bacterial concentration. Meanwhile at 20 min, VUV light disinfection can only result in a minimum of 3-log10 reduction in bacterial count, which is much longer when compared to the *E coli,* ESBL and MRSA experiments described in previous sections. Previous studies [19, 35, 36] showed that mycobacterial species are generally more resistant to UV disinfection, but are subject to a better disinfection effect under VUV light. It seemed that VUV light disinfection was less effective against MTB at a lower bacterial concentration.

### Influenza viruses H1N1 and H3N2

Meanwhile for viral disinfection, test results were considered acceptable when the viral-induced cytotoxic effect is indistinguishable from test agent-induced cytotoxic effects. VUV light disinfection time against viruses would be considered sufficient when a minimum of 3-log10 reduction in viral-induced cytotoxicity in titer was achieved. Therefore, the infectious viruses recovered from the positive controls must be ≥4-log10 for valid viricidal test results. To determine the disinfection efficacy of VUV light against seasonal influenza viruses, two common influenza A viruses, H3N2 and H1N1, causing seasonal epidemics were used. In the current study, initial viral loads for both H1N1 and H3N2, presented in the Additional file 1 as Expt. 9 and Expt. 10, were 5.4 ± 0.4 log10(TCID_50_/mL) and 5.1 ± 0.8 log10(TCID_50_/mL), respectively.

For samples with log10(TCID_50_/mL) less than 1.5, the titer was treated as 0.5 for log reduction calculation and graph plotting purpose.

**Fig. 4:**
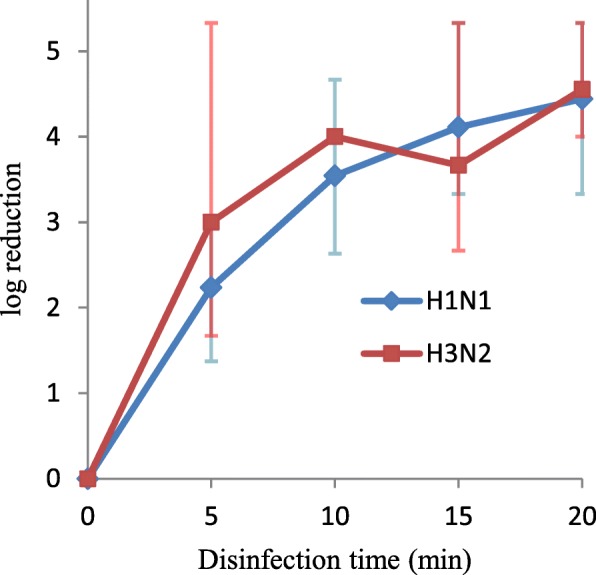
VUV light disinfection against H1N1 and H3N2 influenza A viruses. The log10 (TCID_50_/mL reduction) was plotted against disinfection time

At 5 min of illumination, VUV light can inactivate H1N1 and H3N2 by 2.2- and 3.0-log10 folds viral load (TCID_50_), respectively (Fig. 4). When the VUV illumination time was extended to 20 min, more than 4-log10 reductions in TCID_50_ of both seasonal influenza A viruses were observed.

## Discussion

High-energy vacuum-UV light is efficient in disinfection. Similar to other UV disinfection mechanisms, direct illumination of VUV could result in the formation of new bonds between adjacent nucleotides, causing photochemical damage on DNA strands and eventually inactivating the replication of microorganisms.

In addition, the high-energy VUV could also lead to the formation of both OH radicals and O_3_, which diffuse into anywhere that is shielded from direct UV irradiation and inhibit the growth of microorganism. This explained the excellent bactericidal efficiency of VUV light disinfection even in the presence of the opaque nitrocellulose filter. Our result has further revealed the potential of VUV light to provide a thorough disinfection, even for dust particles and large aerosols contaminated with pathogens where direct UV illumination cannot penetrate.

In this study, we demonstrated that VUV light disinfection is effective against *Escherichia coli*, Extended Spectrum Beta-Lactamase-producing *E. coli* and Methicillin-resistant *Staphylococcus aureus*. For the best tested situation, with the criterion of 3-log10 inactivation of bacteria, valid germicidal result can be achieved with ≤10 min of VUV treatment. Additionally, more than 5-log10 reduction in viable plate count can be attained below 15 min of disinfection.

In the disinfection tests against seasonal influenza viruses H1N1 and H3N2, we also demonstrated that viral load could be effectively reduced by 4-log10 folds after 20 min VUV illumination and this also satisfied the criterion of valid germicidal result. Additionally, more than 3-log10 reduction in viral load can be attained with < 10 min of treatment.

*Mycobacterium tuberculosis*, on the other hand, required a more intense disinfection.

At 20 min disinfection, VUV light disinfection could only result in a 3-log10 reduction in viable plate count. This is insufficient according to our 5-log10 reduction criterion for mycobacterial disinfection. It was only after 30 min of disinfection that the required 5-log10 reduction of *Mycobacterium tuberculosis* viable bacterial load could be achieved regardless of the bacterial concentration. This is concordant to previous studies [19, 35, 36] where mycobacterial species were generally more resistant to UV disinfection. This is probably accounted by the thicker lipid cell wall in *Mycobacterium* species.

The tested variations in concentrations of bacteria did not manifest a trend in the rate of inactivation. For *E. coli* and ESBL, higher bacterial concentration resulted in lower rates of inactivation. Experiments with MTB showed a different trend. Nevertheless, no obvious trend was showed in the experiments with MRSA.

From literature, various research teams reported the UV dosages required attaining 99.9% (3-log) inactivation of various bacteria or viruses under light from low pressure mercury vapour lamps. For example, the UV dosages in mJ/cm^2^ for 3-log inactivation of T7 phage, *E coli.*, *Staphylococcus aureus*, *Mycobacterium avium* and *Mycobacterium phlei* are 10 [37], 5 [37], 9 [34], 18 [20] and 158 [34], respectively. Most of their experiments were conducted with bacteria and viruses virtually unprotected. In our experiment, attaining 3-log inactivation typically required 10 min. Considering that our equipment provided 21 and 2.3 mW/cm^2^ light power at 254 nm and 185 nm, and the total UV power is ~ 23 mW/cm^2^. The UV dosage of 10 min illumination is ~ 14,000 mJ/cm^2^, far higher than the usual values. This could be the consequence of our testing condition created by loading the suspended bacteria or viruses onto nitrocellulose filter paper. Some bacteria were actually protected from direct UV light by the shading effect of filter paper which is different from the testing setup in the literature.

In order to provide sufficient disinfection against all the microorganisms we included in this study, we suggested the use of *Mycobacterium* reduction as a benchmark test for future disinfection instrument designs that incorporates the VUV light system.

Although, the disinfection under the environment with a moderate barrier to light was successful, there are limitations in the present study. The current pilot study on the disinfection efficacy of VUV light disinfection was conducted in laboratory-controlled conditions. For example, due to safety consideration, device type testing on aerosolized bacteria and viruses is not possible. All bacterial and viral inoculums were prepared in liquid suspension and illuminated by VUV on a Petri dish, which differed from actual environmental settings.

## Conclusion

Airborne pathogens are important indoor air quality concerns. A good and reliable disinfection system is a must to maintain good indoor air quality. Vacuum-UV lamps with ozone production were found to be effective for inactivating various human pathogens. With the best tested situation, 3-log10 inactivation of *Escherichia coli*, Extended Spectrum Beta-Lactamase-producing *E. coli*, Methicillin-resistant *Staphylococcus aureus* and seasonal influenza viruses can be achieved with ≤10 min of VUV treatment except *Mycobacterium tuberculosis* which needed about 20 min. This demonstrated the high resistance against UV disinfection of MTB. Valid germicidal results, reflected with 3-log10 inactivation for bacteria, 4-log10 inactivation for viruses and 5-log10 inactivation for MTB, can be obtained with all tested pathogens. The duration of VUV treatment required for valid germicidal result of most of the bacteria was ≤10 min while MTB needed about 30 min. 20 min was adequate for the influenza viruses. This indicated that VUV light is an effective approach against different environmental and pathogenic microorganisms, and can potentially be used for air-purifying units in future ventilation systems.

## Supplementary information

**Additional file 1.** This file contains all data supporting the findings in this study which is a spreadsheet file containing raw data and intermediate calculations.

## Data Availability

All data supporting the findings in this study are contained within the supplementary information files.

## Abbreviations

ATCC: American type culture collection
BHI: Brain heart infusion
CFU: Colony-forming units
CPE: Cytopathic effect
*E. coli*: *Escherichia coli*
ESBL: Extended spectrum beta-lactamase
IAQ: Indoor air quality
MDCK: Madin-Darby canine kidney
MEM: Minimum essential medium
MRSA: Methicillin-resistant *Staphylococcus aureus*
MTB: *Mycobacterium tuberculosis*
O_3_: Ozone
OADC: Oleic acid-albumin-dextrose-catalase
OH: Hydroxyl radical
PBS: Phosphate-buffered saline
TCID_50_: Tissue culture infective dose 50
TPCK: 6-(1-tosylamido-2-phenyl) ethyl chloromethyl ketone
UV: Ultraviolet
UVC: Ultraviolet C
VUV: Vacuum ultraviolet
